# Non-invasive arterial blood pressure measurement and SpO_2_ estimation using PPG signal: a deep learning framework

**DOI:** 10.1186/s12911-023-02215-2

**Published:** 2023-07-21

**Authors:** Yan Chu, Kaichen Tang, Yu-Chun Hsu, Tongtong Huang, Dulin Wang, Wentao Li, Sean I. Savitz, Xiaoqian Jiang, Shayan Shams

**Affiliations:** 1 McWilliams School of Biomedical Informatics, University of Texas Health Science Center at Houston, Houston, TX USA; 2 Institute for Stroke and Cerebrovascular Disease, University of Texas Health Science Center at Houston, Houston, TX USA; 3 Department of Applied Data Science, San Jose State University, One Washington Sq, San Jose, CA 95192 USA

**Keywords:** Deep learning, Blood pressure, Photoplethysmogram, Oxygen saturation, Hypertension assessment, Digital health

## Abstract

**Background:**

Monitoring blood pressure and peripheral capillary oxygen saturation plays a crucial role in healthcare management for patients with chronic diseases, especially hypertension and vascular disease. However, current blood pressure measurement methods have intrinsic limitations; for instance, arterial blood pressure is measured by inserting a catheter in the artery causing discomfort and infection.

**Method:**

Photoplethysmogram (PPG) signals can be collected via non-invasive devices, and therefore have stimulated researchers’ interest in exploring blood pressure estimation using machine learning and PPG signals as a non-invasive alternative. In this paper, we propose a Transformer-based deep learning architecture that utilizes PPG signals to conduct a personalized estimation of arterial systolic blood pressure, arterial diastolic blood pressure, and oxygen saturation.

**Results:**

The proposed method was evaluated with a subset of 1,732 subjects from the publicly available ICU dataset MIMIC III. The mean absolute error is 2.52 ± 2.43 mmHg for systolic blood pressure, 1.37 ± 1.89 mmHg for diastolic blood pressure, and 0.58 ± 0.79% for oxygen saturation, which satisfies the requirements of the Association of Advancement of Medical Instrumentation standard and achieve grades A for the British Hypertension Society standard.

**Conclusions:**

The results indicate that our model meets clinical standards and could potentially boost the accuracy of blood pressure and oxygen saturation measurement to deliver high-quality healthcare.

**Supplementary Information:**

The online version contains supplementary material available at 10.1186/s12911-023-02215-2.

## Background

Chronic cardiovascular and cerebrovascular disease are considered to be among the most prevalent causes of death nowadays [1], and hypertension is one of the leading causes of heart disease and stroke [2]. Hypertension is defined as systolic blood pressure (SBP) higher than 140 mmHg or diastolic blood pressure (DBP) higher than 90 mmHg [3]. According to the World Heart Federation, approximately 50 percent of ischemic strokes are caused by hypertension [4]. In addition, with the Covid-19 pandemic ravaging the world, a recent study shows that almost 75% of patients who have died due to Covid-19 in Italy had a history of hypertension, which indicates a high correlation between hypertension and Covid-19 mortality [5]. For patients who are suffering from vascular diseases, blood pressure (BP) monitoring and management are crucial [6].

Arterial Blood Pressure (ABP) is an invasive continuous BP measurement method that has been widely accepted as the golden standard [7]. However, since the mechanism of ABP is to insert a catheter into an artery to conduct real-time BP monitoring, it is highly sensitive to body movement, such as position changes, and it is relative to the accessed artery, which may bring risks of complications to the patient like infection [8]. Techniques such as cuff-based ABP measurement devices have been widely used for monitoring ABP. However, such measurements might cause discomfort to patients during the inflation and deflation of the cuff. This can affect the accuracy and introduce higher levels of uncertainty [9–11]. Thus, researchers and clinicians are interested in non-invasive approaches [12, 13] to measure ABP.

**Fig. 1:**
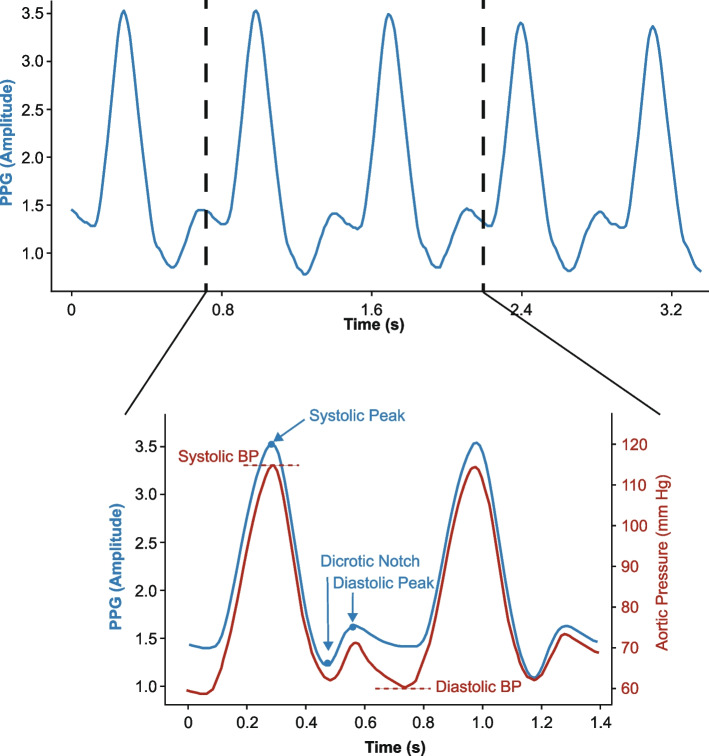
PPG signals (upper) and Aortic Pressure in a beat cycle (lower) from MIMIC III dataset. The top figure presents an example of a PPG signal (blue), and the bottom one represents details of a beat cycle with a corresponding ABP signal (red)

Photoplethysmography (PPG) and electrocardiogram (ECG) signals can be collected by non-invasive measurement devices such as Fiber optic sensors [6] and force-sensitive electromechanical film sensors [14], and have shown a close correlation in estimating ABP. Although both ECG and PPG signals are available in the MIMIC database, ECG signals have a higher missing rate, and the lead count varies for different patients [15]. Moreover, recording ECG signals is generally considered to be more bothersome than recording PPG signals, which is not preferable for a non-invasive ABP estimation method [16, 17]. Additionally, ECG devices are expensive, while fingertip PPG devices are cheap, so there won't be scalability issues [18]. ABP is a quasi-periodic signal in sync with the patient’s heartbeat. The peak in each period refers to arterial systolic blood pressure (ASBP), and the lower bound refers to arterial diastolic blood pressure (ADBP), and correlations between ASBP, ADBP, and PPG signals are shown in Fig. 1.

In recent years, studies have focused on estimating ABP using PPG signals, adopting the spatial–temporal neural network consisting of convolutional neural networks (CNN) and gated recurrent units (GRU) to predict ABP level using PPG signals and its first and second derivative [19]. Athaya et al. employed a U-net-based deep learning architecture to estimate ABP waveforms using PPG signal only, and estimation of related ABP is achieved by using the peak detection algorithm on the MIMIC III dataset [20]. EI Hajj et al. introduced a GRU-based deep learning approach with time domain-based features to estimate ABP using raw PPG signals [21]. Despite the extraordinary performance achieved by deep learning-based methods, their requirement for specific input formats may limit their deployment, as heterogeneity in clinical settings may involve ABP estimation given PPG signals with varying sequence lengths. Furthermore, previous studies have generally been limited in terms of the number of subjects included, and robustness is not fully discussed.

In addition to the possibility of ABP estimation, PPG signals serve as a promising input to estimate oxygen saturation (SpO_2_) with appropriate modeling [22]. SpO_2_ is an indicator of the percentage of hemoglobin saturated with oxygen at the time of the measurement [22, 23]. Normal SpO_2_ ranges from 95 to 100 percent, while below 90 percent is considered abnormal and called hypoxemia, which may cause other complications, such as nausea [24], fatigue [25], organ damage, and failure [26]. The absorption of LED’s green light by oxyhemoglobin and deoxyhemoglobin has significant differences, therefore, the changes of PPG signals induced by blood flow fluctuation can be used to estimate Oxygen saturation level in blood. Several studies have demonstrated how PPG signaling could be used to measure the level of SpO_2_ [22, 27]. However, to the best of our knowledge, no previous study has utilized deep learning (DL) models on PPG signals acquired from medical devices to estimate ABP and SpO_2_ levels simultaneously. These methods failed to estimate ABP and SpO_2_ simultaneously due to noise in signals introduced by ABP and SpO_2_. Here we want to propose a continuous and hardware-independent solution to address the problem of predicting ABP and SpO_2_ using PPG signals and reduce the implementation cost. In addition, a relatively small sample size may introduce higher uncertainty in the deep learning model training process and undermine generalizability.

In [28–30], the methods based on Pulse Transit Time (PTT) were proposed. PTT refers to the time used for ABP waves transiting to the wrist, where the PPG signal is recorded. Moreover, several recent studies introduced additional features based on the second derivative of the PPG signal and demographic features to boost the accuracy of estimating ABP [31–33]. All feature extraction-based methods start with extracting features from the PPG signal with a pre-defined feature extraction pipeline, which may fail to capture all information inherited in the signals and lead to deteriorated generalizability. In addition, none of the feature-extraction-based methods exploit temporal information by considering PPG signals as time series data undermining the estimation performance. Furthermore, diverse machine learning and deep learning-based pipelines were proposed in recent years. Su et al. employed a Long-Short term memory (LSTM) network to PPG signals processed by PTT [34], while Kachuee et al. explored the combination of AdaBoost and PTT on the MIMIC II dataset [35]. Due to the ability to extract local temporal features, a convolutional neural network (CNN) was also utilized in the estimation of ABP using PPG [36–38]. However, these methods may suffer from inflexibility and lack of generalizability due to fixed kernel size, or inefficiency in temporal feature extraction. Therefore, we hypothesize that using Time Series Transformer on PPG signals alone could provide accurate ABP and SpO_2_ prediction for ICU patients.

Hence, in this paper, we propose a method to estimate ABP and SpO_2_ using Transformer-based deep learning architecture. Our model only needs the raw PPG signal to estimate ASBP, ADBP, and SpO_2_. To the best of our knowledge, this is the first work to estimate ASBP, ADBP, and SpO_2_ using PPG signals. This approach has many advantages, including:

1. This method is an end-to-end approach to processing PPG signals and can handle different periods of PPG signals to deliver accurate ABP and SpO_2_ estimation.
2. The model was developed using unsupervised pre-training and supervised fine-tuning. The utilized approaches are less prone to overfitting and are appropriate for personalized model creation with high accuracy.
3. The model satisfies the requirements of the Association of Advancement of Medical Instrumentation (AAMI) standard and achieves grade A for the British Hypertension Society (BHS) standard, with significantly low mean absolute error (MAE) than standard criteria on a large test cohort (1,732 patients in total).
4. This method transforms an invasive measurement of ABP to a non-invasive approach while maintaining high accuracy, therefore, it reduces risks to the patient and is more applicable for continuous monitoring.

## Methods

### Data and preprocessing

Our work utilizes the MIMIC III database, available online per request [39]. This database contains a mixture of different types of digital data (typically contains ECG, ABP, respiration, PPG, and others) with over 60,000 records from more than 30,000 ICU patients. Each patient has at least one record, which ranges from seconds (usually anomalies) to hundreds of hours. Following methods described in the previous study [19], we extracted ABP and PPG signals from bedside waveform (only 10,282 patients have waveform signals documented) records, and cleaned and pre-processed the original data to rule out the anomalies. Considering the impact of the length of signal slices on prediction accuracy, we conducted a literature search and presented it in Supplementary Table 1. We discovered that the chosen sample lengths for different models ranged from beat-wise (approximately 1 to 2 s) to 32 s. Additionally, we performed an initial investigation on the range of heart rates found in the MIMIC III dataset, and identified reference that indicate a minimum heart rate of around 20 bpm [40]. We plotted the histogram of the heart rate distribution in our dataset in Supplementary Fig. 1. Upon analyzing the heart rate distribution, we observed that, as the dataset comprises ICU patients, there are indeed slices with relatively lower heart rates (less than 40 bpm). Given that we were considering the use of a Time Series Transformer for our proposed model, we empirically selected a 20-s sample length. We performed the preprocessing and cleaning steps first, and to further denoise the PPG signal and extract the latent signal for model training, we conducted empirical mode decomposition (EMD) on PPG 20-s slices and divided the results into 4 channels and fed them into a Transformer encoder-based model. The embedding of each record is fed as an input into a feed-forward network for regression on the corresponding SpO_2_, ASBP, and ADBP values.

**Fig. 2:**
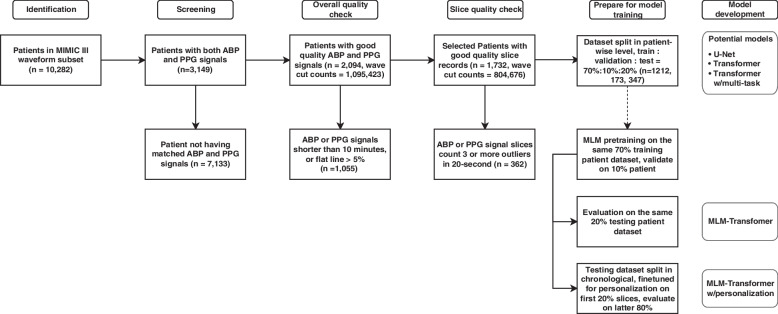
Signal preprocessing flow gram. The figure shows patient/data inclusion/exclusion criteria in each major step

The preprocessing and cleaning steps of the dataset are illustrated in Fig. 2. The entire waveform dataset was downloaded into a dedicated server and transformed to the proper MATLAB format by the wfdb2mat function in the WFDB software package [41]. In addition, using waveform type information from the header files, we excluded those records that do not contain ABP or PPG (specified as ABP and PLETH in header files) and removed the files smaller than 17 kilobytes.

Afterward, we took more detailed cleaning procedures to enhance waveform quality. Firstly, we set the time length threshold of records to be 10 min while records shorter than that were deleted. Then, we checked the morphology of waveform signals and found that a large proportion of signals contain outliers, which can be divided into two major categories: flat lines and flat peaks. We applied a cycle peak-valley detection algorithm proposed by [19, 42] on both ABP and PPG signals, and signals were further cut into slices, which contain separate cycles. Using locations of peaks and valleys, we defined “flat” as 3 or more consecutive equal values; hence, the proportion of flat parts in the whole record of both ABP and PPG signals was calculated. If the proportion was beyond 5%, we simply discarded the entire record. For those records containing flat parts less than 5%, we discarded the flat part and sutured the remaining part.

**Fig. 3:**
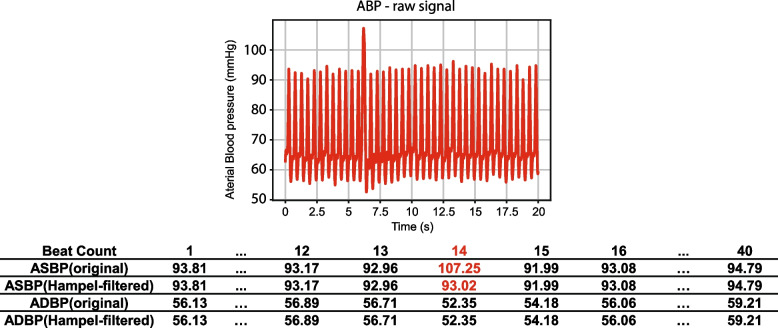
Illustration plot of ABP signal slice, its corresponding ASBP values at peaks before and after Hampel Filtering. A motion artifact is detected and the abnormal ASBP value is replaced with the window average median

**Fig. 4:**
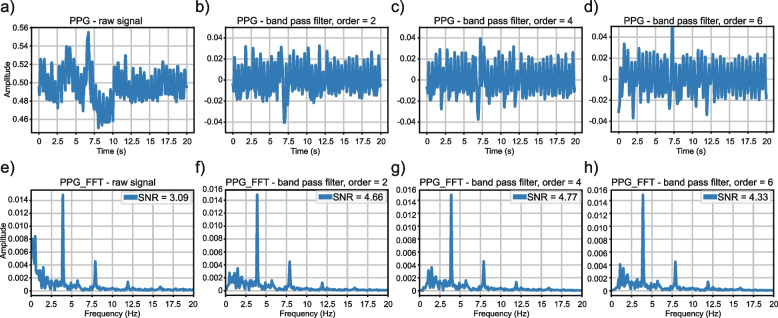
Illustration plot of PPG signal slice and Fast Fourier Transform Frequency Domain after Butterworth Bandpass Filtering. **a**—**d**) present signal morphology before and after Butterworth bandpass filter of orders 2, 4, and 6, respectively, with fixed frequency threshold [0.5, 8] Hz. **e**—**h**) present corresponding Power Spectral Density by Fast Fourier analysis. As subplot (g) shows, noise exists with a lower frequency and is diminished after filtering. SNR: Signal–Noise Ratio

After the initial preprocessing, we filtered the ABP data using a Hampel filter [43] with a sliding window of 100 values. For each window, the median was computed, and outliers were replaced with this median if they fell 3 sigmas below or above it. It is important to note that, as our data came from different patient sources, we did not apply any global or local normalization to the records. As shown in Fig. 3, the Hampel filter effectively filtered abnormal ASBP and ADBP values due to its robustness against outliers. The PPG data was filtered using a 4th-order Butterworth band-pass filter with a frequency range of 0.5 to 8 Hz. This helped to filter out the baseline wander below 0.5 Hz and high-frequency noise above 8 Hz, which can contribute to eliminating motion artifacts and high-frequency noise [44]. A demonstration of the filters applied to the signals is shown in Fig. 4. Fast Fourier Transform frequency analysis revealed several noise peaks in the range of 0.5 to 8 Hz. The 4th-order Butterworth filter was used because it can provide a smooth, flat frequency response in the passband, coupled with a rapid transition to the stopband, without overshoot or ringing. Examples of unsuitable ABP and PPG segments can be found in Supplementary Fig. 2.

We carried out a segmented cycle quality assessment for both ABP and PPG signals. Starting from the initial time point of the cardiac cycle in the signal, we analyzed the subsequent 20-s segments, identifying all beat cycles within this duration using a cycle peak-valley detection algorithm proposed by [19, 42]. If we detected more than 3 anomalous cycles in either the ABP or PPG signals, we disregarded that specific segment. Subsequently, if the segment passed the quality assessment, we computed the average values for ASBP and ADBP using ABP signals. A comprehensive outline of our data selection criteria can be found in Supplementary Table 2.

**Fig. 5:**
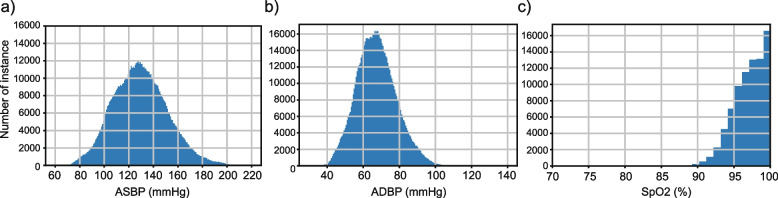
Distribution of (**a**) arterial systolic BP (ASBP), (**b**) arterial diastolic BP (ADBP), and (**c**) SpO_2_ in the processed dataset

After preprocessing and cleaning of the dataset, we have in total 804,676 slices with related ASBP and ADBP values, belonging to 2,641 records of 1,732 patients. The distribution of ASBP, ADBP, and SpO_2_ values is shown in Fig. 5.

To denoise the PPG signals, we employed Empirical Mode Decomposition (EMD) because it captures key features of each heartbeat and provides additional input channels for our TST model. Please refer to Supplementary Fig. 3 for an example plot of EMD applied to PPG signal segments. EMD is particularly suitable for this task as it makes only mild assumptions about time-series data and does not rely solely on simple sine and cosine waves, allowing it to be used for denoising and feature extraction while retaining the original dimensions of the raw signals. We conducted an exploratory experiment based on the MLM-transformer model to determine the optimal number of channels. We tested 2, 3, 4, and 8 channels and found that the model with 4-channel EMD-processed data yielded satisfactory MAE performance (see Supplementary Table 3). Consequently, we used 4-channel time-series data with a dimension of 2,500 (corresponding to 20-s) as our input for model training. Table 1 presents a comparison of prediction performance using different signal denoising techniques, demonstrating that EMD is effective in feature extraction.

**Table 1:** Results of baseline comparison using different signal denoising techniques. Experiments were conducted using a Vanilla Transformer structure trained 70% training patients’ records and evaluated over 30% testing patients’ records

Denoising techniques	SBP (MAE ± S.D., mmHg)	DBP (MAE ± S.D., mmHg)
Original Signal	7.54 ± 8.19	4.02 ± 4.63
Signal + 1^st^ and 2^nd^ derivative	7.16 ± 7.41	3.85 ± 4.12
Wavelet	6.83 ± 5.48	3.54 ± 4.27
**Empirical Mode Decomposition**	**6.76 ± 5.24**	**3.57 ± 4.39**

#### Proposed model

Recently, Transformer-based models achieved superior performance in Natural Language Processing and Computer Vision tasks due to their extraordinary ability of temporal feature extraction and representation [45, 46]. In addition, several previous studies applied Transformer to time series prediction, showing its potential in time-series regressions [47, 48]. Our deep learning architecture utilizes the Transformer encoder [45]. The TST model is based on the attention mechanism, which considers any pair of readings from the input channels with an attention score, while RNN-based models rely on a hidden memory state to store previous information, which is hard to parallelize during model training and may trigger information loss due to improper selection of hidden state dimension, leading to undermined estimation performance [34, 36, 37]. Moreover, the proposed model utilized unsupervised pre-training and supervised fine-tuning strategies for personalized models, which maintains high generalizability in ICU cases. In addition, local patterns extracted by attention mechanisms could boost model performance. The reason we did not adopt the full Transformer architecture with encoders and decoders is that the estimation of ABP and SpO_2_ concentrates on feature extraction from input data instead of signal reconstruction tasks. To be specific, the Transformer encoder maintains scalability in feature extraction from time-series inputs and is more suitable for our tasks. We refer the readers to the original paper of Transformer for details on Transformer encoder architecture for further clarification.

#### Transformer encoder model

For each training sample $X \epsilon {R}^{w\times l} = [{x}_{1}, {x}_{2}, ..., {x}_{l} ]$, where *w* refers to the number of channels generated by EMD, and *l* refers to the length of the channel. Standardization for each channel is conducted by subtracting its mean and dividing by the standard deviation, and then a linear projection onto *D*-dimensional representation space is used, where *D* refers to the input dimension of the transformer model:

1 $${z}_{t} = {W}_{p} \cdot {x}_{t}+{b}_{p}$$

where ${W}_{p} \epsilon {R}^{D\times w}, {b}_{p} \epsilon {R}^{D}$ are learnable linear projection parameters. The output ${z}_{t}$ serves as the input for the transformer encoder model, which could be used as queries, keys, and values in self-attention layers. Without external information, self-attention focuses on the extraction of internal information and latent patterns within signals. Mathematically, the linearly projected signal Z serves as query, key, and value after multiplying three trainable weights vectors ${\omega }^{q}$, ${\omega }^{k}$ and ${\omega }^{v}$, and l represents the length of Z:

2 $$Attention(Q,K,V) = softmax(\frac{Q{K}^{T}}{\sqrt{l}})V$$

where:

$$Q = {\omega }^{q}\cdot Z$$

$$K = {\omega }^{k}\cdot Z$$

$$V = {\omega }^{v}\cdot Z$$

Since the attention mechanism could not naturally capture ordering information, a positional encoder is required to inform the model of the ordering of input signals. We added positional encodings ${W}_{pos}$ to the input vector${z}_{t} \epsilon {R}^{D\times l} = [{z}_{1}, {z}_{2}, ..., {z}_{l}]$:

3 $$Z\mathrm{^{\prime}} =Z +{W}_{pos}$$

**Fig. 6:**
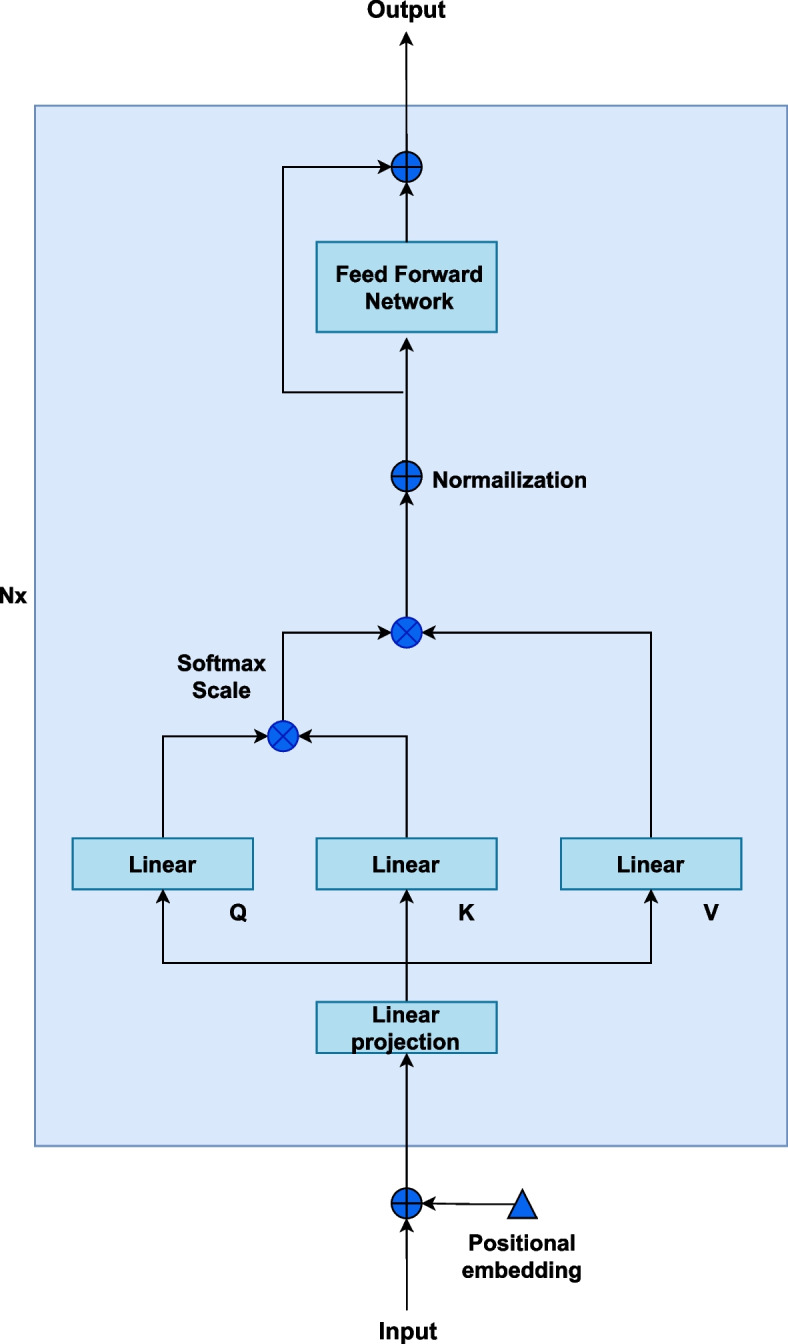
Illustration of self-attention-based encoder structure

We noticed that in the original transformer encoder, the deterministic sinusoidal positional encoder achieved extraordinary performance in many NLP tasks, however, PPG signals maintain periodic patterns, and a learnable positional encoding could extract such patterns more effectively. Although the input dimension and length of PPG signals in our dataset remain fixed, we may encounter considerable variance in signal length when deploying in real life. This issue is effectively solved with our Transformer-based approach: after setting a maximum signal length $l,$ samples with shorter input lengths are padded. The padding process introduces extremely large negative values to the attention scores for the padded positions. In our implementation, therefore, a padding mask is utilized to force the model to overlook padded positions. By doing so, our model could potentially handle any PPG signal shorter than our maximum length. Finally, a layer normalization is conducted after computing self-attention scores and the linear layer of each encoder block, leading to a robust output. The overall encoder structure is shown in Fig. 6.

To deliver regression on ASBP, ADBP, and SpO_2_, we adopt fully connected layers to project embedding vectors learned by transformer encoder blocks onto a scalar value for each task:

4 $$\widehat{y} ={W}_{o}\cdot Z\mathrm{^{\prime}} +{b}_{o}$$

where ${W}_{o}$ and ${b}_{o}$ are learnable parameters in fully connected layers. The loss function is mean squared error $L = \frac{1}{n}\Sigma ||\widehat{y} -y|{|}^{2}$ where $y$ is the ground truth and n is the batch size.

#### Pre-trained and fine-tuning for personalization regression

To further investigate the proposed model in personalized ABP and SpO_2_ prediction, we introduced unsupervised learning-based pre-training and fine-tuning techniques to conduct personalized prediction. The unsupervised pre-training part aims at learning latent patterns inherited in PPG data from training samples of a diverse cohort, while the fine-tuning part conducts personalized ABP and SpO_2_ prediction using individual training samples and model parameters initialized by the pre-training part.

In the unsupervised pre-training part, an autoregressive task is utilized. We borrowed the idea from Masked Language Modeling (MLM) [49], and basically, for each PPG sequence, a random set of records is masked, and the model is asked to predict the masked region of the sequence. Data splitting was performed by random selection of patients into training and testing datasets, and demographic feature statistics of splitting is calculated. For each patient’s record, the model was finetuned with the first 20% as training and the rest 80% as evaluation. This chronological split helps to avoid data leakage. A summary of the dataset splitting was presented in Table 2, and an illustration of dataset splitting was given in Supplementary Fig. 4. We further fine-tuned the pre-trained transformer encoder to personalize the model for each patient. An ablation study was conducted to find the best rate for finetuning to be 20% (please see Supplementary Table 4).

**Table 2:** Summary of the dataset splitting

	Training	Validation	Testing	Testing (Finetune-part)	Testing (Evaluation)
Patient count	1,212	173	347	347	347
Slices count	580,871	74,279	149,527	29,893	119,634
Slices/patient (Mean ± S.D.)	479.26 ± 761.96	429.70 ± 658.27	430.91 ± 632.15	86.15 ± 127.31	344.77 ± 493.6
Min slice count	10	10	10	2	8
Max slice count	6,339	4,507	4,186	837	3349
SBP (mmHg, Mean ± S.D.)	126.26 ± 20.87	125.91 ± 21.94	129.34 ± 22.06	129.58 ± 21.50	129.29 ± 21.15
DBP (mmHg, Mean ± S.D.)	67.49 ± 11.19	67.25 ± 11.34	67.40 ± 11.25	67.36 ± 11.29	67.41 ± 11.13
SpO_2_ (%,Mean ± S.D.)	97.21 ± 2.19	97.16 ± 2.31	97.27 ± 2.19	97.49 ± 2.15	97.22 ± 2.20

To further improve model performance and generalizability, we adopted personalized model training, which considers patients with multiple samples available (at least a 200-s PPG signal available). We split each patient’s samples as training, validation, and testing sets in chronological order, where we use all patients’ samples in the training set to conduct pre-training, which serves as the base model for all patient-specific fine-tuning tasks and fine-tuning on each patient’s sample in the testing set. Then each patient-specific model is validated on the corresponding validation set to determine the best model. The result reported in the result section is evaluated on each patient’s testing set. All models used in this paper are based on a 3-layer Time Series Transformer with ReLU as an activation function. For different tasks, a fully connected layer with hidden dimension 128 was used. We used MLM-Transformer as the pre-training task with 200 epochs and 3e-3 as fine-tuning learning rate with the beta coefficient for learning rate decay after epoch 50. The fine-tuning task will terminate if validation loss could not decrease within 20 epochs (patience parameter as 20).

**Fig. 7:**
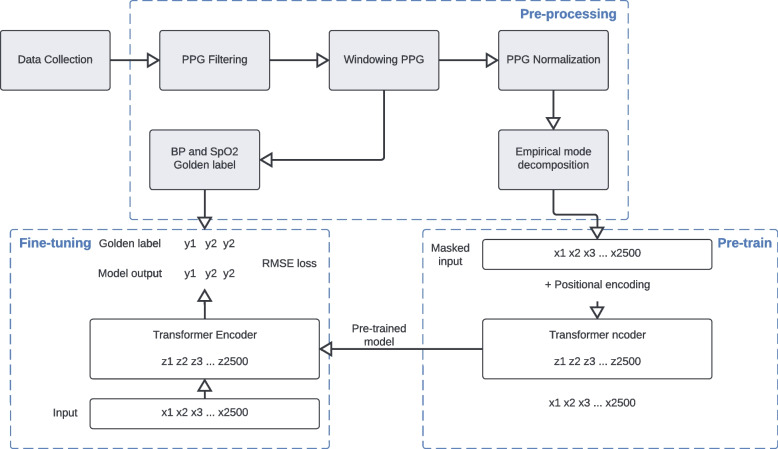
ABP and SpO_2_ Estimation using PPG by Transformer-based Time Series Model

The overall architecture of our Transformer-based model on the estimation of ABP and SpO_2_ using PPG data is shown using a flowchart in Fig. 7.

#### Evaluation metrics and experimental setup

ABP and SpO_2_ prediction is compared with other models by the mean absolute error (MAE), which is defined as

5 $$MAE = \frac{1}{N}{\sum }_{i=1}^{N}||\widehat{y} -y||$$

and the rooted mean square error (RMSE), which is defined as

6 $$RMSE =\sqrt{\frac{1}{N}{\sum }_{i=1}^{N}||\widehat{y}-y|{|}^{2}}$$

where N is the number of record counts in a batch, ŷ is the model prediction and y is the golden label.

#### Training environment

Python v3.9.10 (Python Software Foundation) with package ‘pytorch’ v1.13.0 [50] was used for data processing and model building. All experiments were conducted on DGX-2 and 2 Nvidia v100 GPUs.

## Results

### Estimation of ABP and SpO2

A demographic statistics summary of the dataset used is presented in Table 3.

**Table 3:** Demographic statistics of dataset split. For hypothesis testing, we conducted Welch's two-tailed t-test to compare the means of age and length-of-stay-at-hospital between two independent groups and Pearson’s Chi-square test for other categorical variables. To account for multiple comparisons, *p*-values were adjusted using the Bonferroni Correction. All *p*-values > 0.05 indicate that there is no evidence to suggest that the groups are statistically different or that there is an association between the categorical variables tested

		Missing	Overall	Train	Validation	Test	*p*—value
**Demographic features**			1732	1212	173	347	
Gender, n (%)	Female	0	778 (44.9)	535 (44.1)	129 (49.6)	114 (43.8)	0.255
	Male		954 (55.1)	677 (55.9)	131 (50.4)	146 (56.2)	
Length of stay at hospital, days, mean (SD)		0	10.0 (12.6)	10.1 (13.4)	8.7 (7.9)	10.4 (12.5)	0.192
Age, years, mean (SD)		0	62.0 (58.3)	61.8 (56.4)	64.4 (65.4)	61.0 (59.2)	0.77
Ethnicity, n (%)	Caucasian	0	1206 (69.6)	843 (69.6)	180 (69.2)	183 (70.4)	0.996
	Unknown/Not specified		158 (9.1)	116 (9.6)	25 (9.6)	17 (6.5)	
	African American		134 (7.7)	93 (7.7)	17 (6.5)	24 (9.2)	
Admission type, n (%)	Emergency	0	1174 (67.8)	822 (67.8)	169 (65.0)	183 (70.4)	0.567
	Newborn		287 (16.6)	192 (15.8)	52 (20.0)	43 (16.5)	
	Elective		228 (13.2)	166 (13.7)	32 (12.3)	30 (11.5)	
Insurance, n (%)	Private	0	761 (43.9)	526 (43.4)	115 (44.2)	120 (46.2)	0.612
	Medicare		733 (42.3)	509 (42.0)	116 (44.6)	108 (41.5)	
	Medicaid		152 (8.8)	112 (9.2)	22 (8.5)	18 (6.9)	
Religion, n (%)	Catholic	18	555 (32.4)	390 (32.5)	78 (30.4)	87 (33.7)	0.357
	Not specified		369 (21.5)	249 (20.8)	64 (24.9)	56 (21.7)	
	Unobtainable		273 (15.9)	181 (15.1)	50 (19.5)	42 (16.3)	
Marital status, n (%)	Married	363	682 (49.8)	472 (48.6)	99 (49.7)	111 (56.1)	0.338
	Single		370 (27.0)	276 (28.4)	49 (24.6)	45 (22.7)	
	Widowed		198 (14.5)	139 (14.3)	37 (18.6)	22 (11.1)	

To compare the performance of the proposed approach, several baselines (basic transformer and CNN-based U-Net) are adopted for the prediction of ABP and SpO_2_, which are shown in Table 4. Additionally, we performed an ablation study using a 5-s sample length, which led to MAE and SD values for ASBP and ADBP of 2.52 ± 2.63 and 1.37 ± 1.89, respectively.

**Table 4:** Overall prediction performance (measured as MAE) of the proposed Transformer Models (bottom 3) on the test dataset for ASBP, ADBP, and SpO_2_ in comparison with traditional Transformer and U-Net model as baseline. MLM, fine-tuned with Masked Language Modeling

Methods	Parameter Counts, × 10^6^	SpO_2_, %	ASBP, mmHg	ADBP, mmHg
Transformer	1.4	1.65 ± 1.94	6.76 ± 5.24	3.57 ± 4.39
U-Net	13.3	1.02 ± 1.46	5.03 ± 4.78	2.98 ± 3.41
Transformer w/multi-task	2.2	1.28 ± 1.67	6.44 ± 5.32	3.42 ± 4.17
MLM-Transformer	2.2	0.75 ± 1.04	4.97 ± 4.72	2.99 ± 2.39
**MLM-Transformer w/personalization**	**2.2**	**0.56 ± 0.79**	**2.41 ± 2.72**	**1.31 ± 1.77**

For the multi-task Transformer model, we fine-tuned the pre-trained Transformer model for the regression of ASBP, ADBP, and SpO_2_ simultaneously, which showed undesirable performance due to heterogeneity among ABP and SpO_2_ readings of ICU patients.

**Fig. 8:**
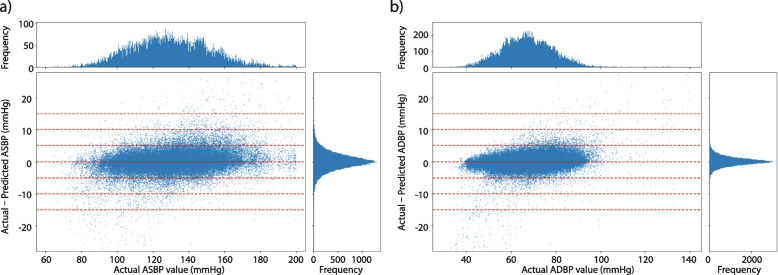
Bland–Altman scatterplot for (**a**) ASBP and (b) ADBP values. The top histograms present golden label distributions for the test set, and the right histograms present the distributions of differences between predicted and actual values

**Fig. 9:**
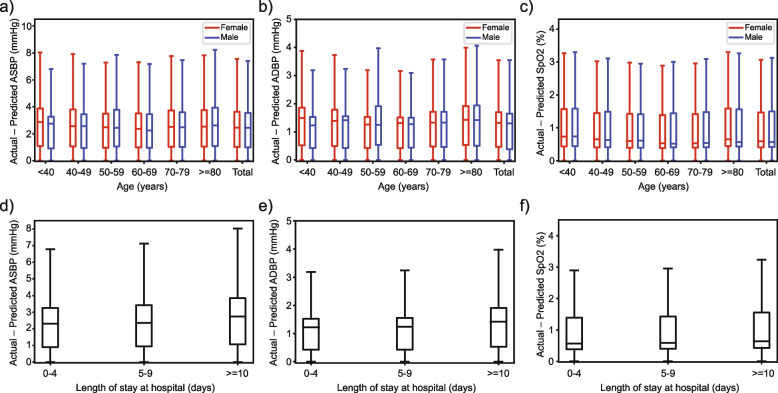
Model performance on demographic subgroups. **a**—**c**) present the performance of patients of different ages and gender, and **d**—**f**) present the performance of patients with different lengths of stay at the hospital. Columns from left to right: ASBP, ADBP, and SpO_2_. As plot shows, our proposed model does not present significant prediction differences in different subgroups

The Bland–Altman plots for the personalized Transformer model are shown in Fig. 8. The x-axes refer to pressure from 60 to 220 mmHg for ASBP and 30 to 140 mmHg for ADBP, while the y-axes stand for error ranging from -30 to 30 mmHg for our estimation. The dashed horizontal lines refer to error at -15 to 15 mmHg with 5 mmHg as intervals step size. As Fig. 8 shows, most of the ASBP and ADBP errors lie in [-5, 5]. A subgroup analysis of prediction performance was conducted, based on the patients’ gender, age, and length of stay at hospitals, and the results are shown in Fig. 9. No demographic prediction bias was found considering these features.

### Comparison with state-of-the-art studies

As demonstrated in Table 5, the results of our approach were compared with other state-of-the-art studies on PPG-BP predictions. The evaluation metrics used are estimation error (MAE ± S.D.) and the squared Pearson correlation coefficient (r^2^) for systolic blood pressure (SBP) and diastolic blood pressure (DBP). We achieved 2.41 ± 2.72 and 1.31 ± 1.77 for SBP and DBP with *r*^2^ = 0.982 and *r*^2^ = 0.971, respectively, which is better than the existing approaches in BP estimation.

**Table 5:** Comparison of methods and result of state-of-the-art studies with proposed model

	Data source	Method	Personalized	Metrics	SBP	DBP
(Su et al. 2018) [34]	Proprietary data (84 subjects, 10 min each)	PTT + deep learning (LSTM)	Unknown	RMSE	3.73	2.43
(Mohammad Kachuee et al. 2017) [35]	MIMIC II (1000 subjects)	PTT + classical ML (AdaBoost)	Yes	MAE	11.17	5.35
(Kurylyak, Lamonaca, and Grimaldi 2013b) [51]	MIMIC (15,000 beats)	Temporal PPG features + artificial neural network (ANN))	Unknown	MAE	3.8	2.21
(Gupta et al. 2022) [52]	MIMIC I, II, and III (39 subjects, ? subjects, 510 subjects)	PPG signal’s derivative contours + ML algorithms	Yes	MAE	0.74, 1.69, 1.30	0.35, 0.77, 0.56
(Bernard, Msigwa, and Yun 2022) [36]	MIMIC II (69 subjects) and proprietary data (23 subjects)	five 1-D CNN, three Bi-directional LSTM networks	No	MAE	1.38	0.95
(Leitner, Chiang, and Dey 2022) [37]	MIMIC III (100 subjects)	RCNN neural networks + personalization	Yes	MAE	3.52	2.2
(Wang et al. 2022) [53]	MIMIC II (348 records)	Visibility graph + transfer learning	No	MAE	6.17	3.66
(Schlesinger et al. 2020) [38]	MIMIC II (304 subjects)	CNN + Siamese Network	No	MAE	5.95	3.41
Our proposed work (MLM-Transformer w/personalization)	MIMIC III (1,732 subjects)	3-layer Time Series Transformer + personalized fine-tuning	Yes	MAE	2.41	1.31

We compared our proposed transformer-based model with feature extraction-based ML or DL models. The recent study from Gupta et al. used nonlinear features extracted from the third and fourth derivatives of the PPG cycle, combined with random forest as one of the traditional machine-learning models [52]. The authors reported achieving 1.30 ± 4.05 and 0.56 ± 1.70 for SBP and DBP, respectively, on the MIMIC III dataset. However, a selected smaller sample (510 subjects) was used in that study and the denoising process was not fully presented.

Moreover, the time sequence consistency between slices was not taken into consideration in the dataset splits, which can sabotage the validity of the results. In addition, the inflexibility of the input format may undermine its potential in a real-world deployment. Traditional models such as CNN-RNN combined deep learning model can indeed improve prediction performance through fine-tuning techniques, which is demonstrated by Leitner et al. [37]. Through personalization, the MAE in that study improved from 4.59 and 2.72 mmHg for SBP and DBP to 3.52 and 2.20 mmHg respectively. However, we think that the natural setting of the fixed count of convolutional channels and the dimension of the convolutional kernel results in limited applicability to PPG signals with motion artifacts and heterogeneity of the patient cohort. And in the compared study, the qualified patients have 10-h high-quality data. Also, in the compared study, there is no control of time sequence in fine-tuning, namely the model can be trained on later slices and evaluated on earlier slices. On the other hand, please kindly note that our inclusion criteria require only 10 or more segments of qualified slices which are 200 s in length in total, and we used all available samples from the fine-tuned patient record. Moreover, we used the earlier 20% slices for fine-tuning and the later 80% for evaluation to avoid data leakage problems.

On the other hand, Su et al. utilized ECG and PPG signals with Long Short-Term Memory (LSTM) networks to predict BP waveform, which achieved RMSE of SBP and DBP of 3.73 and 2.43, respectively [34]. However, it’s based on healthy subjects whose SBP and DBP maintain lower fluctuation compared with ICU patients. Moreover, some works based on the MIMIC II dataset were proposed. Kurylvak's group initially introduced the neural network to handle PPG signals of the MIMIC II dataset, which illustrated the potential of a neural network-based approach in signal processing of vital signs [51]. Kachuee et al. proposed an Adaboost-based framework to process vital signs, which achieved moderate performance due to model simplicity and small sample size [51].

### Compliance with standards

The prediction on the test dataset is compared with the AAMI error standard for the estimation of ABP [54]. Note that the AAMI standard requires an ME less than 5 in the prediction of ABP with more than 85 subjects (we also presented MAE as a more rigorous criterion which is commonly used in previous PPG-BP prediction work) A comparison between the AAMI standard and our proposed model is shown in Table 6.

**Table 6:** Comparison of our results with the Association for the Advancement of Medical Instrumentation (AAMI) standard

		No. of Subjects	MAE (mmHg)	ME (mmHg)	STD (mmHg)
AAMI Standard ^30^		> 85	/	< 5	< 8
MLM-Transformer	ASBP	347	4.97	0.043	4.72
	ADBP	347	2.99	0.026	2.39
MLM-Transformer w/personalization	ASBP	347	**2.41**	**0.037**	**2.72**
	ADBP	347	**1.31**	**0.029**	**1.77**

In addition, the performance of MLM-Transformer w/personalization is also evaluated by the British Hypertension Society (BHS) grading standard [55], which is shown in Table 7. The BHS grading standard measures the cumulative percentage of a pre-defined error range. According to the evaluation, our results achieved “Grade A”, which means at least 60% of samples with less than 5 mmHg error, at least 85% of samples with less than 10 mmHg error, and at least 95% of samples with less than 15 mmHg error.

**Table 7:** Comparison of our results with the British Hypertension Society (BHS) grading standard

		Cumulative Error (%)		
		≤ 5 mmHg	≤ 10 mmHg	≤ 15 mmHg
BHS grading standard [55]	Grade A	60%	85%	95%
	Grade B	50%	75%	90%
	Grade C	40%	65%	85%
MLM-Transformer w/personalization	ASBP	89.73%	97.61%	99.24%
	ADBP	97.33%	99.62%	99.86%

## Discussion

In this study, we developed a novel end-to-end transformer-based deep learning model for the prediction of ABP and SpO_2_ using a PPG signal and evaluated its performance on the MIMIC III waveform dataset. Results show that our model reached the prediction accuracy as MAE of ASBP, ADBP, and SpO_2_ by 2.41, 1.31, and 0.56, respectively, and complies with current standards for ABP prediction. The proposed model is trained and evaluated on 1,732 patients and over 4000 records in total which to the best of the author’s knowledge, is the largest subset compared to former studies.

As shown in Fig. 5, after preprocessing, we collected PPG and ABP signal data from 1,732 subjects, which is much higher compared with previous studies. Although we achieved similar performance with [20], our model is capable of delivering ABP and SpO_2_ prediction simultaneously with pre-trained and fine-turned models, which serve as a satisfactory personalization model not only for ICU patients but also for more wide-scale general usage. It is worth noting that utilizing preprocessing steps we proposed, we produced a subset of the MIMIC III waveform subset with high-quality PPG signal slices with ABP-based blood pressure as golden labels. It should be noted that such preprocessing steps could filter out a large proportion of raw signal data due to a lack of synchronized ABP or SpO_2_ signals, or relatively low-quality PPG signals with nuances, such as flat lines and fluctuations.

To fully utilize all available data, we further implemented unsupervised pre-training which enables us to train personalized models using not only golden-labeled data, but all training samples to deliver robust and reliable results. Due to the large data dimension and heterogeneity among patients, especially ICU patients, feature extraction-based methods are impractical and hard to implement in real life [56]. Hence, we chose deep learning models to deliver end-to-end ABP and SpO_2_ prediction. Different from LSTM and other recurrent neural networks, transformer-based models do not require complicated gates and cell designs to process sequential information. Instead, an attention mechanism is utilized to automatically recognize significant patterns from raw data, which not only boosts the model’s ability in latent pattern recognition but also saves computational resources. In addition, since the transformer model was proposed naturally to process unstructured text data, it could tolerate different lengths of input by introducing masks to pad the input.

In our experiment, the boost of prediction accuracy was achieved through supervised personalization, which is essentially fine-tuning a pre-trained transformer model with the upstream slices of the same patient to predict downstream tasks. This further increased the MAE of ASBP, ADBP, and SpO_2_ by 4.35, 2.26, and 1.09, respectively. We used rather unconstrained inclusion/exclusion criteria when filtering a subset from the MIMIC III waveform subset regarding the patient’s demographic and clinical information, such as ICD-9 diagnosis, to ensure the wide range of its application. Therefore, it is expected to generate a rather heterogeneous subset over the original one. Such heterogeneity in clinical conditions has been reported to connect with different ABP clinical impact and management strategies [57], in addition to demographic features (impact of different age, BMI, gender, and race/skin color for PPG absorption as an example [58]). Thus, we anticipated a personalization of the model would help further improve the prediction performance.

As shown in Fig. 2, the prediction performance remains relatively stable within a large span of ABP distribution. We also compared the results with AAMI and BHS standards and results show that the MLM-Transformer w/personalization complies with the standards for ensuring accuracy, safety, and effectiveness, meanwhile the model also achieved satisfied prediction accuracy for SpO_2_ prediction. It is worth noting that in this dataset, the distribution of patients’ SpO_2_ is highly skewed, and only 0.6% and 15.5% of our samples are considered below standards, using thresholds of 90% and 95% respectively. Predicting SpO_2_ levels with accuracy and conductibility has gained more attention in recent years, especially during Covid-19 pandemic [59, 60]. One potential future study would be transferring and evaluating this model on the Covid-19 patient dataset.

This study has several limitations. One major drawback of using PPG signals to predict blood pressure is the potential for data bias. PPG signals are highly sensitive to changes in skin color and commonalities, which can vary significantly from person to person (Wang et al., 2016). As a result, the PPG data collected may not be representative of the general population and may be biased toward certain demographic groups or patient groups. Due to data accessibility, the relationship between comorbidities and the prediction performance of the proposed model was not studied, which remains for future research. Another limitation of this approach is its generalizability. The MIMIC III dataset is a collection of records from ICU patients, so the applicability of the proposed model to other cohorts and healthy subjects requires further investigation on external datasets. MIMIC-II is considered by its authors an older version of MIMIC-III (and MIMIC-IV https://mimic.mit.edu/docs/ii/), and also there is an issue of overlapping patients between different versions of the MIMIC database (https://github.com/MIT-LCP/mimic-code/issues/229). A potential hazard of data leakage may occur if we evaluate the model trained on MIMIC-III to MIMIC-II, so we decided not to do so. Although there was a lack of external evaluation, we trained and tested our model on sample slices acquired from 1,732 subjects, of which cohort size is expanded compared to former studies. We will continue evaluating our work in clinical settings in future studies. Finally, there are potential issues in the deployment of the proposed personalized model, which can be computationally expensive, and we plan to further investigate how to put the proposed personalization method into practice. In future studies, a combination of additional information, e.g., demographic features such as age and gender, clinical information, and deep learning model may further improve the model performance. Moreover, properly defined loss functions considering the heterogeneity may boost the performance of a multi-task Transformer. Lastly, data utilized in this study was solely collected from ICU patients [39], and incorporating this MIMIC III waveform subset with a dataset collected from healthy people may help further increase the prediction performance.

## Supplementary Information

**Additional file 1:** **Supplementary Figure 1.** Heart Rate distribution in the preprocessed dataset and 10–60 bpm subset **Supplementary Figure 2.** Illustration plot of unqualified slices of signals. a - b) unqualified ABP signal slices, and c - d) unqualified PPG signal slices **Supplementary Figure 3.** Illustration plot of Empirical Model Decomposition results of PPG signal slices after preprocessing **Supplementary Figure 4.** Demonstration plot of dataset splitting inpatient-wise level or with fine tuning **Supplementary Table 1.** A literature search results of the relationship between the length of signal slices and the size of the dataset **Supplementary Table 2.** Presentation of the basis of the data selection. For context, please refer to ‘Data and preprocessing’ in the Methods section **Supplementary Table 3.** The exploratory experiment result of prediction comparison for the number of channels used in EMD. **Supplementary Table 4.** Ablation study results.

## Data Availability

The data that support the findings of this study is part of the original MIMIC III waveform matched subset, which was used under license for the current study and is publicly available upon permission (research proposal requested), and is available at: https://physionet.org/content/mimic3wdb-matched/1.0/ [39].

## Abbreviations

AAMI: Association of Advancement of Medical Instrumentation
ABP: Arterial Blood Pressure
ADBP: Arterial diastolic blood pressure
ASBP: Arterial systolic blood pressure
BHS: British Hypertension Society
BP: Blood pressure
CNN: Convolutional neural network
DL: Deep learning
ECG: Electrocardiogram
EMD: Empirical mode decomposition
MLM: Masked Language Modeling
GRU: Gated recurrent units
ICU: Intensive care unit
MAE: Mean absolute error
NLP: Natural language processing
PTT: Pulse transit time
SpO_2_: Oxygen saturation
