# Supplementary material for: Non-invasive arterial blood pressure measurement and SpO2 estimation using PPG signal: a deep learning framework
Source: BMC Med Inform Decis Mak. 2023 Jul 21;23:131. doi: 10.1186/s12911-023-02215-2 (PMC10362790; doi:10.1186/s12911-023-02215-2)
Supplement: Supplementary file 1 — Additional file 1: Supplementary Figure 1. Heart Rate distribution in the preprocessed dataset and 10–60 bpm subset Supplementary Figure 2. Illustration plot of unqualified slices of signals. a - b) unqualified ABP signal slices, and c - d) unqualified PPG signal slices Supplementary Figure 3. Illustration plot of Empirical Model Decomposition results of PPG signal slices after preprocessing Supplementary Figure 4. Demonstration plot of dataset splitting inpatient-wise level or with fine tuning Supplementary Table 1. A literature search results of the relationship between the length of signal slices and the size of the dataset Supplementary Table 2. Presentation of the basis of the data selection. For context, please refer to ‘Data and preprocessing’ in the Methods section Supplementary Table 3. The exploratory experiment result of prediction comparison for the number of channels used in EMD. Supplementary Table 4. Ablation study results. [file 12911_2023_2215_MOESM1_ESM.docx]

**Supplementary**

| Title | Dataset(No. subjects) | Number of Segments (total durations) | Number of seconds per Segment |
| --- | --- | --- | --- |
| [(Su et al., 2018)](https://arxiv.org/pdf/1705.04524.pdf) | Static BP Dataset (N = 96) | 2475(22h) | 32s |
| [(Slapničar et al., 2019)](https://www.mdpi.com/1424-8220/19/15/3420) | MIMIC III (N = 510) | 504000(700h) | 5s |
| [(Athaya & Choi, 2021)](https://www.mdpi.com/1424-8220/21/5/1867) | MIMIC III (N = 100) | 195h | 2.048s |
| [(El Hajj & Kyriacou, 2020)](https://openaccess.city.ac.uk/id/eprint/25046/1/) | MIMIC II (N = 85) | 21000 and 9000 | 3s and 7s |
| [(Miao et al., 2017)](https://ieeexplore.ieee.org/stamp/stamp.jsp?arnumber=7914684) | Self-collected dataset (N = 73) | 10 min for each subject | Beat-to-Beat |

**Supplementary Table 1.** A literature search results of the relationship between the length of signal slices and the size of the dataset.

| Variables | Data type | Data source | Frequency | Record length requirement | Flat line | Abnormal beat check | Filtering | Value threshold |
| --- | --- | --- | --- | --- | --- | --- | --- | --- |
| PPG signal | Biomedical signal | Waveform subset | Downsample to 125 Hz | >10 minutes | <5% | <3 count in a slice | Butterworth Bandpass filter ([0.5, 8] Hz, order = 4) | N/A |
| ABP signal | Biomedical signal | Waveform subset | Downsample to 125 Hz | >10 minutes | <5% | <3 count in a slice | Hampel filter (window of 5) of ASBP and ADBP values | ASBP in [60, 200] mmHg  ADBP in [30, 140] mmHg |

**Supplementary Table 2.** Presentation of the basis of the data selection. For context, please refer to ‘Data and preprocessing’ in Methods section.

| # of channels in EMD | SpO_2_ | ASBP | ADBP |
| --- | --- | --- | --- |
| 1 (not applicating EMD) | 1.68 | 7.31 | 3.96 |
| 2 | 0.97 | 5.14 | 3.03 |
| 3 | 0.81 | 4.83 | 2.88 |
| **4** | **0.75** | **4.86** | **2.81** |
| 8 | 0.73 | 4.91 | 2.79 |

**Supplementary Table 3.** Exploratory experiment result of prediction comparison (measured as MAE) for number of channels used in EMD.

| Experimental settings | | SBP (MAE ± S.D., mmHg) | DBP (MAE ± S.D., mmHg) |
| --- | --- | --- | --- |
| Finetuning set % | Evaluation set % |  |  |
| 10 | 90 | 3.76±3.91 | 2.27±2.56 |
| 20 | 80 | 2.41±2.72 | 1.31±1.77 |
| 30 | 70 | 2.53±2.90 | 1.36±1.87 |
| 50 | 50 | 2.42±2.75 | 1.30±1.83 |
| 70 | 30 | 2.19±2.51 | 1.27±1.69 |

**Supplementary Table 4**. Results of ablation study.

**Supplementary Figure 1.** Heart Rate (bpm) distribution in the (a) preprocessed dataset and (b) 10 – 60 bpm subset.

**Supplementary Figure 2.** Illustration plot of unqualified slices of signals. a - b) unqualified ABP signal slices, and c - d) unqualified PPG signal slices.

**Supplementary Figure 3.** Illustration plot of Empirical Model Decomposition results of PPG signal slices after preprocessing.

**
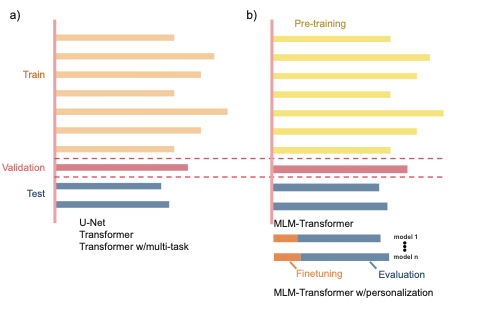
**

**Supplementary Figure 4.** Demonstration plot of dataset splitting in (a) patient-wise level or (b) with finetuning.
